# Preoperative vitamin D deficiency and postoperative delirium risk: multicenter retrospective study

**DOI:** 10.3389/fnut.2025.1617670

**Published:** 2025-07-17

**Authors:** Kuo-Chuan Hung, Ting-Sian Yu, Shu-Wei Liao, Yi-Chen Lai, Pei-Han Fu, Ping-Hsun Feng, I-Wen Chen

**Affiliations:** ^1^Department of Anesthesiology, Chi Mei Medical Center, Tainan City, Taiwan; ^2^School of Medicine, College of Medicine, National Sun Yat-sen University, Kaohsiung City, Taiwan; ^3^Department of Anesthesiology, E-Da Hospital, I-Shou University, Kaohsiung City, Taiwan; ^4^Center of General Education, Chia Nan University of Pharmacy and Science, Tainan City, Taiwan; ^5^Department of Anesthesiology, Chi Mei Medical Center, Liouying, Tainan City, Taiwan

**Keywords:** postoperative delirium, vitamin D deficiency, musculoskeletal surgery, propensity score matching, cognitive dysfunction, perioperative risk factors

## Abstract

**Background:**

To assess the impact of preoperative vitamin D deficiency (VDD) on the risk of postoperative delirium (POD) in patients undergoing musculoskeletal surgery.

**Methods:**

This cohort study utilized the TriNetX Healthcare Commercial Organizations database. We included patients aged 50 years or older who underwent musculoskeletal surgery requiring hospital admission. Patients were categorized according to their preoperative vitamin D levels into three groups: deficient (≤20 ng/mL), insufficient (21–29 ng/mL), and sufficient (≥30 ng/mL). The primary outcome was POD within 30 days of surgery. Secondary outcomes included risks of surgical site infections, emergency department (ED) visits, and intensive care unit (ICU) admissions. Risk factors for POD were assessed using multivariate logistic regression analysis.

**Results:**

After matching, 6,218 pairs of vitamin D-deficient and sufficient patients were compared. VDD was related to a significantly higher risk of POD [1.0% vs. 0.5%; odds ratio (OR): 2.18, 95% confidence interval (CI): 1.41–3.36, *p* < 0.001]. Vitamin D-deficient patients also had higher rates of ED visits (OR: 1.36, 95% CI: 1.18–1.57, *p* < 0.001) and ICU admissions (OR: 1.51, 95% CI: 1.19–1.91, *p* < 0.001). Similarly, vitamin D insufficiency (10,764 matched pairs) was associated a smaller but significant increase in delirium risk (OR: 1.49, 95%CI: 1.05–2.12, *p* = 0.023), along with increased ED visits (OR: 1.16, 95% CI: 1.03–1.30, *p* = 0.013) and ICU admissions (OR: 1.25, 95% CI: 1.03–1.52, *p* = 0.0261), suggesting a dose-dependent relationship. Risk factor analysis revealed that advanced age, male sex, chronic kidney disease, and malnutrition were significant predictors of POD in patients with VDD.

**Conclusion:**

Individuals with VDD experienced a higher risk of POD, suggesting the potential benefits of preoperative vitamin D screening and supplementation as a strategy to improve outcomes in surgical patients. While our findings highlight the potential benefit of vitamin D assessment and optimization before surgery, the retrospective design limits the ability to draw causal inferences. Prospective interventional studies are warranted to determine whether treating VDD can meaningfully lower the risk of POD.

## 1 Introduction

Postoperative delirium (POD) involves fluctuating attention, impaired thinking, and changes in awareness. In lower-risk elective procedures, such as joint replacement, POD occurs in approximately 1–22.8% of cases (1, 2). However, in high-risk settings—such as cardiac procedures—the incidence rises sharply to 4.1–54.9% (3). This acute change in mental status is associated not only with prolonged hospital stays and higher healthcare costs (4) but also with increased mortality and long-term cognitive decline (5–7), potentially leading to reduced quality of life, loss of independence, and heightened caregiver strain. Notably, research indicates that up to 54% of POD cases may be preventable through targeted interventions (8, 9), highlighting the critical need to identify and address modifiable risk factors. Although numerous risk factors for POD have been established, including advanced age, pre-existing cognitive impairment, depth of anesthesia, and specific surgical procedures (10–12), the role of modifiable nutritional factors remains incompletely understood. As the surgical population continues to age, recognizing risk factors and implementing effective prevention strategies has become increasingly essential.

Vitamin D deficiency (VDD) is gaining recognition as a possible driver of numerous negative health outcomes. Beyond its well-established role in bone metabolism, vitamin D exhibits significant neurosteroid properties and influences numerous neurological processes (13, 14). Recent evidence indicates that vitamin D receptors are broadly expressed throughout the central nervous system, especially in regions involved in cognitive function and behavioral regulation (15). Furthermore, VDD has been linked to various neurological conditions, including cognitive decline and dementia (16, 17). Despite the biological plausibility of the association between vitamin D status and postoperative neurological complications, the association of preoperative VDD with POD are poorly characterized. Previous studies examining this association have been limited by small sample sizes, single-institution designs, and inadequate control of confounding factors (18–20). Additionally, the potential dose-dependent effects of vitamin D levels on POD have not been adequately evaluated. In an effort to better characterize this relationship, we performed a retrospective analysis across multiple institutions using data from the TriNetX Healthcare Commercial Organizations database. We proposed that preoperative VDD independently contributes to a higher risk of POD and other complications.

## 2 Methods

### 2.1 Data sources

This study utilized data from the TriNetX database, which containing de-identified patient data from over 140 healthcare institutions. Through the TriNetX platform, researchers had access to real-time de-identified electronic health records, including demographic information, ICD-10-coded diagnoses, procedural data (CPT/ICD-10-PCS codes), medication records, laboratory test results, and clinical observations. All data were fully de-identified in accordance with the Health Insurance Portability and Accountability Act (HIPAA) Privacy Rule using standardized, certified processes before researcher access. The TriNetX platform also maintains robust data governance through secure access protocols, institutional agreements, and continuous audit trials to protect patient privacy. In accordance with the Declaration of Helsinki, this study was approved by the Institutional Review Board of Chi Mei Medical Center (approval no. 11310-E04), which also waived the requirement for informed consent.

### 2.2 Inclusion and exclusion criteria

We included patients aged 50 years or older who underwent musculoskeletal surgery (e.g., arthroscopy, femur and knee joint surgery, and spine surgery) requiring hospital admission. According to preoperative vitamin D status within 4 weeks before surgery, patients were classified into a VDD group (≤20 ng/mL) and a control group (≥30 ng/mL). Individuals with any vitamin D measurement >20 ng/mL in the VDD group or <30 ng/mL in the control group within 1 month before surgery were excluded. Additional exclusion criteria included pre-existing neurological or psychiatric diseases documented within 1 year before surgery (including dementia, Alzheimer’s disease, Parkinson’s disease, schizophrenia, bipolar disorders, substance use disorders, and any documented cognitive impairment), history of cerebrovascular events or brain disorders within 3 months before surgery (including stroke, intracranial hemorrhage, and other brain disorders), and recent critical illness within 1 month before surgery [including sepsis and intensive care unit (ICU) admission] (21).

### 2.3 Propensity score matching

The propensity score was calculated by incorporating demographic characteristics [e.g., age at index, body mass index (BMI), race, sex], preoperative comorbidities (e.g., hypertension and diabetes mellitus), and laboratory data (e.g., hemoglobin). To minimize potential confounding due to protein-bound vitamin D levels (22), serum albumin also was included in the propensity score matching process. In TriNetX, data on vitamin D supplementation, such as prescription records, dosing, and patient compliance, are often unavailable or inconsistently documented. Consequently, we did not use vitamin D supplementation status as a matching variable. Instead, we used serum 25-hydroxyvitamin D [25(OH)D] levels as the primary exposure variable, based on the rationale that measured serum concentrations more accurately reflect patients’ physiological vitamin D status than prescription data alone. In addition, while preoperative cognitive status is an important risk factor for delirium, standardized cognitive assessments are not routinely performed before surgery. Thus, we were unable to match or adjust for this variable because of low availability. A 1:1 nearest neighbor propensity score matching was conducted without replacement, applying a caliper of 0.1 standard deviations of the logit-transformed score.

### 2.4 Study outcomes

We defined the primary outcome as the occurrence of POD within 30 days after surgery, as indicated by ICD-10 codes F05 or R41.0. Secondary outcomes included postoperative complications, such as emergency department (ED) visits, ICU admission, and surgical site infections (ICD-10: T81.41 or ICD-10: T81.42). All outcomes were assessed from the time of surgery to the 30-day postoperative period.

### 2.5 Risk factors assessment

A multivariate logistic regression analysis was performed to evaluate risk factors for POD, incorporating demographic variables (age, sex, race) and preoperative comorbidities such as hypertension and diabetes mellitus. For continuous variables, such as age, odds ratios were calculated per unit increase. We performed separate analyses for the vitamin D-deficient and sufficient groups to identify potentially different risk patterns between these populations.

### 2.6 Additional analysis

An additional analysis was conducted on patients with vitamin D insufficiency (20–30 ng/mL) to explore a potential dose-dependent relationship. Using identical inclusion and exclusion criteria, we compared vitamin D-insufficient patients with matched controls with sufficient levels. This analysis evaluated whether the relationship between VDD and POD follows a dose-dependent pattern across deficient, insufficient, and sufficient levels.

In addition to our primary matching model, we conducted two sensitivity analyses to further validate our findings. In Model I, we restricted the analysis to patients aged ≥65 years to account for age-related vulnerability to delirium. In Model II, we included C-reactive protein (CRP), a commonly used inflammatory marker, as an additional covariate in the matching process to evaluate the role of systemic inflammation.

### 2.7 Statistical analysis

Mean ± standard deviation was used to summarize continuous variables; categorical variables were presented as numbers and percentages. To compare propensity score-matched groups, we calculated odds ratios (OR) with 95% confidence intervals (CI). For risk factor assessment, we conducted separate multivariate logistic regression analyses for the vitamin D-deficient and vitamin D-sufficient groups. In these analyses, we estimated the ORs for each independent variable, including continuous variables such as age, for which the OR was calculated per unit increase. We performed 1:1 nearest-neighbor matching without replacement. This approach is widely accepted as a conservative threshold to reduce bias while retaining an adequate sample size. Matching without replacement was chosen to maintain the integrity of individual matches and avoid repeated controls. To assess the adequacy of matching, we calculated standardized mean differences (SMDs) for all covariates, with values below 0.1 indicating acceptable balance. A plot of SMDs before and after matching was used to visually confirm covariate balance. Statistical analyses were carried out utilizing the TriNetX Analytics platform. A two-sided *p*-value of less than 0.05 was considered indicative of statistical significance across all analyses.

## 3 Results

### 3.1 Characteristics of patients

**Figure 1:**
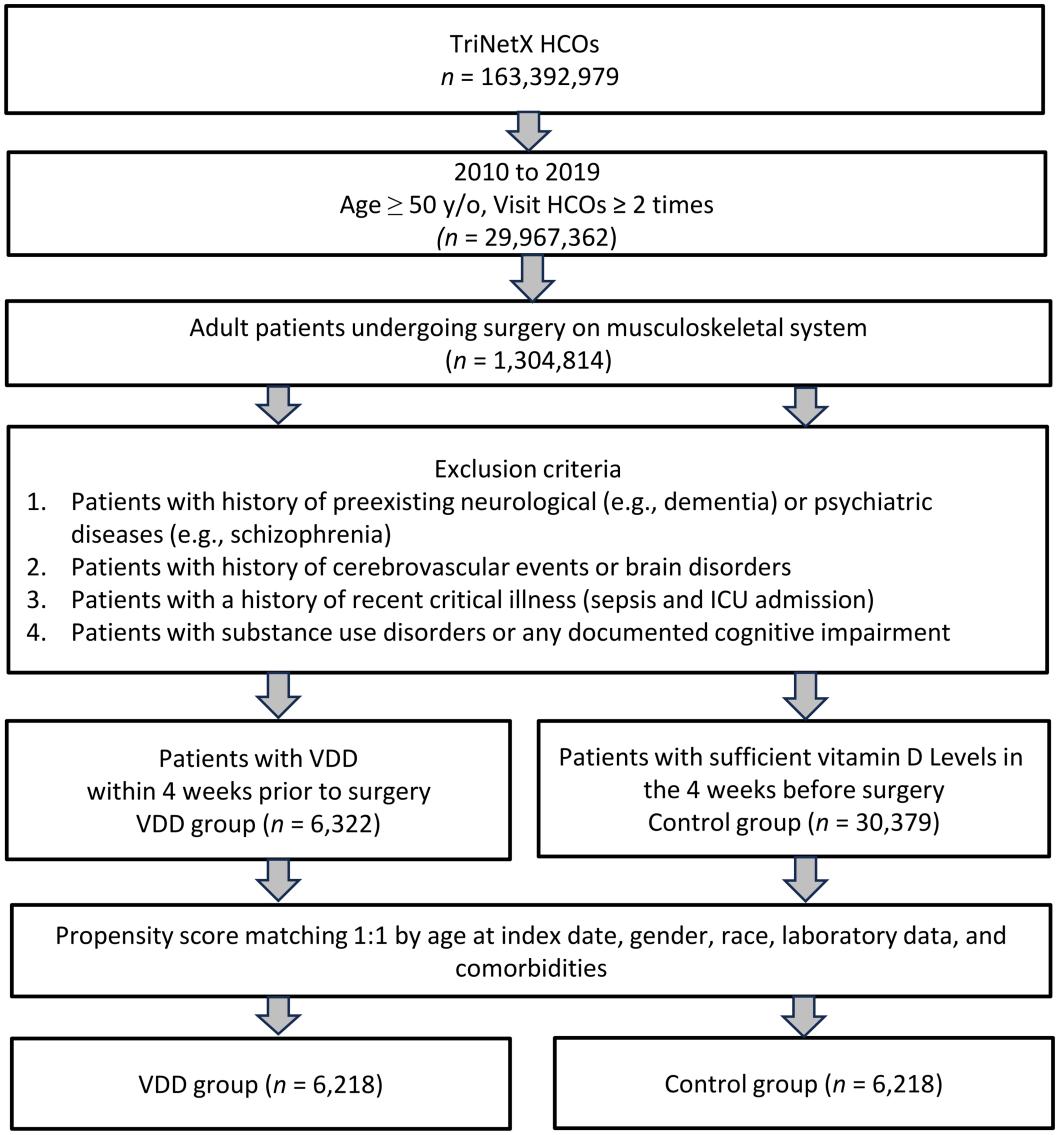
This flowchart illustrates the selection process for the study cohort. HCOs, Healthcare Commercial Organizations; ICU, Intensive Care Unit; PSM, Propensity Score Matching; VDD, Vitamin D Deficiency.

**Figure 2:**
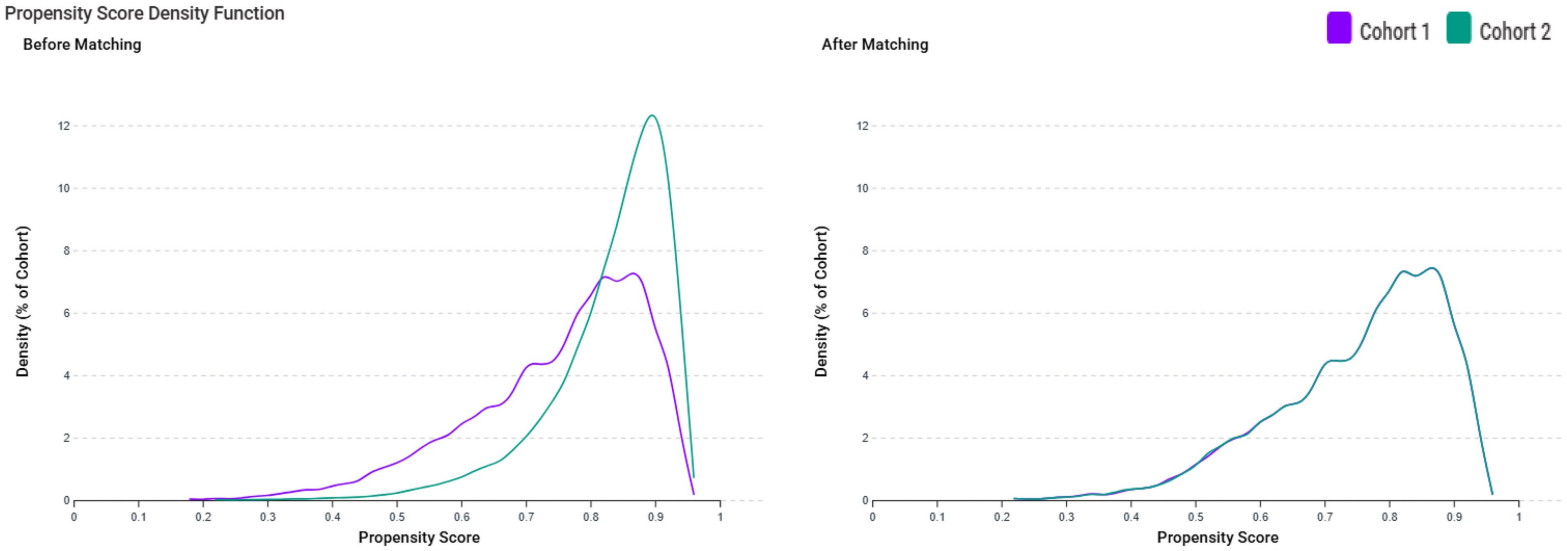
Propensity score density function before and after matching between patients with vitamin D deficiency (VDD) (Cohort 1, purple line) and those without VDD (Cohort 2, green line). The left panel displays the distribution of propensity scores before matching, showing a lack of overlap between the groups. The right panel shows the distribution after matching, demonstrating improved balance between the cohorts.

In this study, 6,322 patients with VDD and 30,379 patients without VDD were identified before matching (Figure 1). After matching, 6,218 pairs were analyzed in the study (Table 1). The balance between cohorts before and after matching was assessed using SMDs for all baseline covariates and visualized using propensity score density plots (Figure 2). The density plots demonstrate substantial improvement in the overlap between groups post-matching, indicating a good balance and appropriate use of the matching algorithm. The mean age was 63.4 years in both groups. Both groups had similar proportions of females (60.7%), and racial distribution was comparable with that of predominantly White patients (68.4%). BMI distributions were similar between the groups (51.9% vs. 51.9% with BMI ≥ 30 mg/kg). Regarding comorbidities, the matched groups showed a similar prevalence of essential hypertension (57.7%), diabetes mellitus (31.5%), neoplasms (26.5%), and ischemic heart disease (17.9%). Other clinical characteristics, including cerebrovascular diseases (6.4%), liver diseases (6.4%), and chronic obstructive pulmonary disease (6.7%), were also well balanced between groups.

**Table 1:** Characteristics of patients with or without vitamin D deficiency.

Variables	Before PSM			After PSM		
	VDD group (*n* = 6,322)	Control group (*n* = 30,379)	SMD	VDD group (*n* = 6,218)	Control group (*n* = 6,218)	SMD
Patient characteristics						
Age	63.2 ± 11/6	67.7 ± 10.6	0.406	63.4 ± 11.6	63.4 ± 10.8	0.005
BMI (≥30 mg/kg)	3,284 (51.9%)	13,280 (43.7%)	0.165	3,225 (51.9%)	3,331 (51.9%)	0.034
Female	3,807 (60.2%)	21,467 (70.7%)	0.221	3,773 (60.7%)	3,758 (60.7%)	0.005
White	4,271 (67.6%)	25,054 (82.5%)	0.350	4,256 (68.4%)	4,252 (68.4%)	0.001
Black or African American	1,246 (19.7%)	2,396 (7.9%)	0.348	1,176 (18.9%)	1,211 (18.9%)	0.014
Asian	118 (1.9%)	795 (2.6%)	0.051	118 (1.9%)	103 (1.9%)	0.018
Health-related service utilization factors	5,192 (82.1%)	25,790 (84.9%)	0.075	5,115 (82.3%)	5,137 (82.3%)	0.009
Underlying health conditions						
Essential hypertension	3,650 (57.7%)	18,359 (60.4%)	0.055	3,588 (57.7%)	3,649 (57.7%)	0.020
DM	2022 (32.0%)	7,171 (23.6%)	0.188	1958 (31.5%)	1902 (31.5%)	0.019
History of neoplasms	1,653 (26.1%)	10,548 (34.7%)	0.187	1,647 (26.5%)	1,630 (26.5%)	0.006
History of ischemic heart diseases	1,143 (18.1%)	5,310 (17.5%)	0.016	1,115 (17.9%)	1,066 (17.9%)	0.021
Chronic kidney disease	1,055 (16.7%)	4,546 (15.0%)	0.047	1,024 (16.5%)	966 (16.5%)	0.025
COPD	424 (6.7%)	2060 (6.8%)	0.003	416 (6.7%)	369 (6.7%)	0.031
Cerebrovascular diseases	403 (6.4%)	2,140 (7.0%)	0.027	397 (6.4%)	339 (6.4%)	0.040
Liver diseases	399 (6.3%)	2093 (6.9%)	0.023	396 (6.4%)	357 (6.4%)	0.026
Malnutrition	230 (3.6%)	602 (2.0%)	0.100	207 (3.3%)	218 (3.3%)	0.010
Hyperparathyroidism and other disorders of parathyroid gland	130 (2.1%)	963 (3.2%)	0.070	128 (2.1%)	99 (2.1%)	0.035
Nicotine dependence	134 (2.1%)	349 (1.1%)	0.077	127 (2.0%)	112 (2.0%)	0.018
Alcohol related disorders	34 (0.5%)	134 (0.4%)	0.014	33 (0.5%)	34 (0.5%)	0.002
Laboratory data						
Hemoglobin (≥12 g/dL)	4,734 (74.9%)	25,370 (83.5%)	0.214	4,695 (75.5%)	4,710 (75.5%)	0.006
Albumin g/dL (≥3.5 g/dL)	4,266 (67.5%)	24,068 (79.2%)	0.268	4,244 (68.3%)	4,264 (68.3%)	0.007

COPD, Chronic obstructive pulmonary disease; DM, Diabetes mellitus; PSM, propensity score matching; VDD, vitamin D deficiency; BMI, body mass index; SMD, standardized mean differences.

### 3.2 Postoperative clinical outcomes in individuals with VDD

The incidence of POD was significantly higher in patients with VDD (1.0%) than in patients without VDD (0.5%) (OR: 2.18, 95%CI: 1.41–3.36, *p* < 0.001) (Table 2). This finding indicates that VDD is related to more than double the odds of POD development. Among the secondary outcomes, we observed significant differences in healthcare utilization between the two groups. ED visits within 30 days after surgery were more frequent in vitamin D-deficient patients (7.3% vs. 5.5%, OR 1.36, 95%CI: 1.18–1.57, *p* < 0.001). Similarly, ICU admission risk was significantly higher in vitamin D-deficient patients (OR: 1.51, 95%CI 1.19–1.91, p < 0.001). Regarding postoperative complications, risk of surgical site infection was comparable between the two groups (OR: 0.83, 95%CI 0.42–1.65, *p* = 0.601).

**Table 2:** Primary and secondary outcomes at 30-day follow-up in patients with vitamin D deficiency.

Outcomes	VDD group (*n* = 6,218)	Control group (*n* = 6,218)	OR (95% CI)^‡^	*p*-value
	Events (%)	Events (%)		
Postoperative delirium	65 (1.0%)	30 (0.5%)	**2.18 (1.41, 3.36)**	<0.001
Surgical site infection	15 (0.2%)	18 (0.3%)	0.83 (0.42, 1.65)	0.601
ICU admission	176 (2.8%)	118 (1.9%)	**1.51 (1.19, 1.91)**	<0.001
ED visit	454 (7.3%)	340 (5.5%)	**1.36 (1.18, 1.57)**	< 0.001

VDD, vitamin D deficiency; ICU, intensive care unit; ED, Emergency department. ^‡^Control group as reference. OR, odds ratio; CI, confidence interval. Values highlighted in bold represent statistically significant findings.

### 3.3 Association of vitamin D insufficiency with postoperative outcomes

In our matched cohort analysis of 10, 764 pairs of individuals with vitamin D insufficiency, we observed several significant associations between vitamin D insufficiency and postoperative outcomes. The risk of POD was higher in patients with vitamin D insufficiency than in those with sufficient levels (OR: 1.49, 95% CI 1.05–2.12, *p* = 0.023) (Table 3). While this association was notable, the magnitude of the effect was less pronounced than that observed in the vitamin D-deficient group, suggesting a possible dose-dependent relationship. The analysis of healthcare utilization revealed consistent patterns in vitamin D-sufficient patients. ED visits were also more frequent among patients with vitamin D insufficiency (OR: 1.16, 95%CI:1.03–1.30, *p* = 0.013), as were ICU admissions (OR 1.25, 95%CI: 1.03–1.52, *p* = 0.026). The magnitude of the effect was less pronounced than that observed in the vitamin D-deficient group. Similarly, surgical site infection rates were identical between the two groups (OR: 1.00, 95%CI: 0.62–1.62, *p* = 1.000).

**Table 3:** 30-day outcomes in patients with vitamin D insufficiency.

Outcomes	VDI group (*n* = 10,764)	Control group (*n* = 10,764)	OR (95% CI)^‡^	*p*-value
	Events (%)	Events (%)		
Postoperative delirium	79 (0.7%)	53 (0.5%)	**1.49 (1.05, 2.12)**	0.023
Surgical site infection	33 (0.3%)	33 (0.3%)	1.00 (0.62, 1.62)	1.000
ICU admission	231 (2.1%)	186 (1.7%)	**1.25 (1.03, 1.52)**	0.026
ED visit	670 (6.2%)	585 (5.4%)	**1.16 (1.03, 1.30)**	0.013

VDI, vitamin D insufficiency; ICU, intensive care unit; ED, Emergency department. ‡Control group as reference. odds ratio, OR; CI, confidence interval; VDI, vitamin D insufficiency. Values highlighted in bold represent statistically significant findings.

### 3.4 Sensitivity analysis

In Model I (*n* = 4,049 per group) (Table 4), VDD continued to show a significant association with elevated POD risk (OR: 2.09, 95%CI: 1.32–3.31, *p* = 0.001) and ED visits (OR: 1.26, 95%CI: 1.05–1.50, *p* = 0.012). The associations between ICU admission and surgical site infection were not statistically significant in this older subgroup.

**Table 4:** Sensitivity analyses of 30-day postoperative outcomes in patients with vitamin D deficiency.

Outcomes	Model I		Model II	
	OR (95% CI)	*p*-values	OR (95% CI)	*p*-values
Postoperative delirium	**2.09 (1.32–3.31)**	0.001	**2.26 (1.49–3.44)**	<0.001
Surgical site infection	1.00 (0.42–2.41)	1.000	0.92 (0.52–1.62)	0.773
ICU admission	1.29 (0.99–1.68)	0.060	**1.66 (1.32–2.10)**	<0.001
ED visit	**1.26 (1.05–1.50)**	0.012	**1.30 (1.13–1.50)**	<0.001

VDI, vitamin D insufficiency; ICU, intensive care unit; ED, Emergency department; ‡control group as reference; OR, odds ratio; CI, confidence interval; VDI, vitamin D insufficiency. Values highlighted in bold represent statistically significant findings. Model I, Analysis restricted to patients aged ≥65 years. Model II, Analysis includes C-reactive protein (CRP) as a covariate in propensity score matching.

In Model II, which incorporated C-reactive protein (CRP) as a covariate in the matching process, VDD significantly elevated the risk of POD (OR: 2.26, 95%CI: 1.49–3.44, *p* < 0.001), ICU admission (OR: 1.66, 95%CI: 1.32–2.10, p < 0.001), and ED visits (OR: 1.30, 95%CI: 1.13–1.50, *p* < 0.001) (Table 4). Surgical site infection was not significantly associated with the studied variable (OR: 0.92, 95%CI: 0.52–1.62, *p* = 0.773). These findings support the robustness of our results and indicate that the observed associations persist even after accounting for advanced age and systemic inflammation.

### 3.5 Risk factors for POD in patients with VDD

Multivariate analysis of vitamin D-deficient patients revealed several risk predictors (Table 5). Age (OR: 1.05, *p* < 0.001) and male sex (OR: 1.89, *p* = 0.014) were associated with higher risks. Among comorbidities, chronic kidney disease emerged as a strong risk factor (OR: 2.81, *p* < 0.001), along with malnutrition (OR: 2.59, *p* = 0.017).

**Table 5:** Precipitating factor for postoperative delirium in patients with or without VDD.

	Vitamin D deficiency		Sufficient vitamin D levels	
Variables	OR (95% CI)†	*P*-value	OR (95% CI)†	*P*-value
Age at Index	**1.05 (1.03, 1.08)**	**<0.001**	**1.02 (1.00, 1.03)**	**0.034**
Male	**1.89 (1.14, 3.15)**	**0.014**	0.88 (0.65, 1.19)	0.396
White (yes vs. no)	1.83 (0.95, 3.53)	0.071	**0.55 (0.41, 0.75)**	**<0.001**
Essential (primary) hypertension (yes vs. no)	1.47 (0.79, 2.74)	0.228	0.84 (0.59, 1.20)	0.336
Neoplasms (yes vs. no)	0.76 (0.43, 1.34)	0.346	1.15 (0.87, 1.51)	0.337
Diabetes mellitus (yes vs. no)	0.77 (0.44, 1.36)	0.366	**1.41 (1.05, 1.91)**	**0.023**
Nicotine dependence (yes vs. no)	0.57 (0.06, 5.20)	0.616	0.69 (0.23, 2.03)	0.497
Ischemic heart diseases (yes vs. no)	1.04 (0.58, 1.87)	0.890	**2.51 (1.83, 3.43)**	**<0.001**
Chronic kidney disease (CKD) (yes vs. no)	**2.81 (1.59, 4.99)**	**<0.001**	**2.76 (2.03, 3.76)**	**<0.001**
Alcohol-related disorders (yes vs. no)	0.71 (0.02, 22.33)	0.843	1.14 (0.21, 6.13)	0.883
Cerebrovascular diseases (yes vs. no)	1.60 (0.81, 3.19)	0.178	**2.49 (1.80, 3.45)**	**<0.001**
Chronic obstructive pulmonary disease (yes vs. no)	1.14 (0.52, 2.48)	0.748	**1.96 (1.39, 2.76)**	**<0.001**
Malnutrition (yes vs. no)	**2.59 (1.18, 5.66)**	**0.017**	**2.50 (1.54, 4.06)**	**<0.001**

OR, odds ratio; CI, confidence interval. †Multivariate logistic regression analysis. Values highlighted in bold represent statistically significant findings.

### 3.6 Risk factors for POD in patients without VDD

Multivariate analysis among patients without VDD highlighted multiple independent predictors of POD (Table 5). Advanced age also revealed a significant relationship (OR: 1.02, *p* = 0.034), but White patients demonstrated a protective effect (OR: 0.55, *p* < 0.001). Among comorbidities, chronic kidney disease exhibited the strongest association (OR: 2.76, *p* < 0.001), followed by ischemic heart disease (OR: 2.51, *p* < 0.001), malnutrition (OR: 2.50, *p* < 0.001), and cerebrovascular disease (OR: 2.49, *p* < 0.001). Additional significant risk factors included chronic obstructive pulmonary disease (OR: 1.96, *p* < 0.001) and diabetes mellitus (OR: 1.41, *p* = 0.023).

## 4 Discussion

This large-scale, multi-institutional study revealed that preoperative VDD doubled the risk of POD compared with vitamin D-sufficient patients (1.0% vs. 0.5%). This study also demonstrated a dose-dependent relationship, as vitamin D insufficiency showed a smaller but significant increase in delirium risk (0.7% vs. 0.5%). Secondary outcomes indicated higher rates of ED visits and ICU admissions in both the vitamin D deficient and insufficient groups than in the controls, although the effect was more pronounced in the deficient group. Risk factor analysis highlighted distinct patterns across groups. Among vitamin D-deficient patients, significant predictors of POD included increasing age, male sex, chronic kidney disease, and malnutrition. In contrast, while age, CKD, and malnutrition remained risk factors in vitamin D-sufficient patients, additional predictors emerged, including ischemic heart disease, cerebrovascular disease, COPD, and diabetes mellitus.

Our findings revealed a strong link between preoperative VDD and POD, supported by both biological mechanisms and clinical evidence. Vitamin D, functioning as a neurosteroid, influences the central nervous system by helping regulate neurotransmitter activity, reduce oxidative stress, and modulate neuroinflammatory processes (23, 24). Delirium can be triggered by oxidative stress, neuroinflammation, and neurotransmitter imbalances, particularly acetylcholine deficiency and excess dopamine (25). In animal models, vitamin D reduced neuroinflammation by modulating cytokines (26) and helped restore neurotransmitter balance by boosting acetylcholine synthesis (27) and regulating dopamine levels (28). However, much of the mechanistic evidence stems from experimental or preclinical studies, and the direct relevance of these pathways in perioperative settings remains uncertain. Therefore, while these mechanisms provide a possible explanatory framework, they should be interpreted with caution and considered hypothesis generating rather than conclusive. The perioperative period is particularly vulnerable to vitamin D metabolism. Studies have shown that serum 25(OH)D levels can decrease substantially following surgery, potentially due to increased cellular uptake during tissue regeneration and impaired postoperative generation of the active form 1,25(OH)2D (29, 30). This decline in vitamin D levels, combined with the metabolic stress of surgery, may contribute to an increased risk of delirium. The dose-dependent relationship observed between vitamin D status and delirium outcomes aligns with previous findings, which suggest a dose-dependent neuroprotective effect of vitamin D (31, 32). Our findings suggest that vitamin D supplementation is a potentially modifiable factor for delirium prevention. Further research is needed to establish precise preoperative target levels and develop evidence-based supplementation protocols.

In our previous meta-analysis of 2,673 patients (33), we found that preoperative VDD significantly increased the risk of POD and cognitive dysfunction (OR = 1.54). However, the limited sample size (33) prompted us to conduct this large-scale, multi-institutional, retrospective study to further evaluate the association between vitamin D status and POD. Our current findings were largely consistent with the meta-analysis (33), demonstrating that patients with VDD experienced increased risk of POD, though with a stronger association (OR 2.18 vs. 1.54). This stronger effect size in our current study might be attributed to our larger sample size and better control for confounding factors. Interestingly, our previous meta-analysis (33) did not identify a significant association between vitamin D insufficiency and POD. This discrepancy is likely due to the limited sample size in the meta-analysis (33), which included only four studies and 1,410 patients for vitamin D insufficiency analysis, whereas our current study examined a significantly larger cohort.

The increased rates of ED visits and ICU admissions among vitamin D-deficient patients suggest that vitamin D status may have broader implications for postoperative recovery beyond cognitive outcomes. Our findings may partially align with those of a previous study of 872 patients undergoing hip fracture surgery, which reported that low vitamin D levels were associated with an increased risk of medical readmissions within 30 days (34). A notable finding was the dose-dependent relationship; while both vitamin D deficient and insufficient patients showed higher rates of ED visits and ICU admissions compared to those with sufficient levels, the magnitude of the effect was substantially larger in the deficient group. These findings suggest that low vitamin D levels may compromise postoperative recovery and increase the need for higher levels of care. Interestingly, we found no significant difference in surgical site infection rates between the groups. This contrasts with previous studies suggesting a role for vitamin D in immune function and wound healing (35, 36). The absence of a significant association may reflect the low overall rate of surgical site infections in our cohort, which likely reduced the statistical power to identify meaningful differences.

Among vitamin D-deficient patients, significant predictors of POD included increasing age, male sex, chronic kidney disease, and malnutrition. This age-related vulnerability aligns with a previous review article that consistently identified advanced age as a risk factor for POD (37). Male sex was identified as a significant risk factor for POD among vitamin D-deficient patients in the current study. This result aligns with earlier research identifying male sex as a contributing risk factor for POD (38, 39). However, in patients with sufficient vitamin D levels, male sex did not appear to be associated with an increased risk of POD in our study. This novel observation suggests that adequate vitamin D levels might help mitigate certain risk factors associated with male sex. Chronic kidney disease and malnutrition exhibited a notably strong association with delirium in both groups, indicating that these predictors were independent of vitamin D status. While previous studies have established links between these factors and POD (40, 41), our study, with its large sample size, provides robust evidence that vitamin D levels exert minimal influence on these associations. Additional risk factors, such as ischemic heart disease, cerebrovascular disease, chronic obstructive pulmonary disease, and diabetes mellitus, were identified exclusively in vitamin D-sufficient patients. This implies that VDD may alter the impact of traditional risk factors on the development of delirium.

The interpretation of these findings should take into account several notable limitations of the study. First, as a retrospective observational study, our analysis can only demonstrate associations and cannot establish causality between preoperative VDD and POD. Second, although propensity score matching was applied, key variables were unavailable in the TriNetX database, including preoperative cognitive status, perioperative vitamin D supplementation, seasonal variation, duration of surgery, and intraoperative factors, such as anesthesia depth, pain management, and medication use (e.g., anticholinergics and benzodiazepines). These unmeasured factors may have contributed to the residual confounding. Third, an additional limitation of this study is the reliance on ICD-10 diagnostic codes (F05, R41.0) to identify POD cases. Although administrative coding allows for large-scale analysis, it is subject to variability in clinical recognition and documentation practices, which may lead to underreporting or misclassification, particularly in cases of hypoactive or transient delirium. This limitation may result in the underestimation of the true incidence of POD. Validation against standardized clinical tools, such as the Confusion Assessment Method (CAM), would improve diagnostic accuracy, but such information was not available in the TriNetX database. Furthermore, the database structure prevented us from examining the relationship between specific vitamin D levels and delirium severity. Fourth, the database lacks granular information about critical perioperative factors, such as surgical duration and depth of anesthesia, which could significantly impact delirium risk. Fifth, patients with vitamin D insufficiency were excluded from the main analysis to ensure a clear comparison between well-defined deficient and sufficient groups. However, we acknowledge that excluding this intermediate group may introduce selection bias and limit generalizability. Additionally, because the data were retrospectively retrieved, information on vitamin D supplementation during the perioperative period was not available. These factors may affect the accuracy of group classification and outcome interpretation. Finally, we focused exclusively on patients undergoing musculoskeletal surgery to reduce heterogeneity and enhance internal validity. However, this narrow focus limits the generalizability of our findings to other surgical populations, such as those undergoing cardiovascular, thoracic, or abdominal procedures, which may involve different perioperative risk profiles and delirium mechanisms. Future studies should investigate whether the observed association between preoperative VDD and POD holds across diverse surgical specialties.

## 5 Conclusion

Our findings demonstrate that preoperative VDD is independently associated with an increased risk of POD and adverse healthcare utilization outcomes following musculoskeletal surgery. However, because this was a retrospective observational study, causal relationships could not be inferred. The dose-dependent link between vitamin D levels and postoperative complications highlights the possible value of assessing and correcting vitamin D status before surgery. The distinct risk factor profiles in patients with or without VDD highlight the need for tailored preventive strategies. Future trials should incorporate standardized delirium assessments, detailed perioperative data, and longitudinal vitamin D measurements to evaluate whether preoperative optimization of vitamin D status (e.g., vitamin D supplementation) can reduce delirium risk and improve postoperative outcomes.

## Data Availability

The raw data supporting the conclusions of this article will be made available by the authors, without undue reservation.
